# Determining the Minimal Clinically Important Difference in Somatic Symptom Scale-8 Score in Patients With Somatic Symptoms and Related Disorders: A Six-Month Follow-Up Study

**DOI:** 10.31083/AP46109

**Published:** 2025-08-28

**Authors:** Kazuaki Hashimoto, Takeaki Takeuchi, Noriko Takeda, Akiko Koyama, Masahiro Hashizume

**Affiliations:** ^1^Department of Psychosomatic Medicine, Toho University School of Medicine, 143-8541 Tokyo, Japan

**Keywords:** minimal clinically important difference, somatoform disorders, sensitivity, somatic symptoms, specificity

## Abstract

**Background/Objective:**

Whether changes in Somatic Symptom Scale-8 (SSS-8) scores adequately reflect subjective improvement in patients with somatic symptoms and related disorders (SSRD) at follow-up is unclear. The minimal clinically important difference (MCID) is a criterion of estimating clinically significant improvement derived from patients’ responses to anchor questions that accurately reflect changes in their condition. This study aimed to clarify the MCID value of the SSS-8 for SSRD.

**Methods:**

Patients with SSRD aged 18 to 84 years who attended a university hospital outpatient department in Japan were eligible. The participants were assessed using the SSS-8 for physical symptoms. After approximately 6 months of outpatient treatment, the participants were reassessed using the SSS-8 for physical symptoms. The primary endpoint was the Patient Global Impression of Change score. The secondary endpoint was the physical function items of the Multidimensional Patient Impression of Change questionnaire. These questionnaires were used to define improvements in subjective symptoms as the anchor to estimate the MCID. Receiver operating characteristic analysis was performed based on the anchor questions and the MCID values of the SSS-8 were calculated.

**Results:**

Ninety participants were included. The primary endpoint MCID value for the SSS-8 was –6 points, with an area under the curve (AUC) of 0.87, 65.9% sensitivity, and 93.5% specificity. The secondary endpoint MCID value for the SSS-8 was –6 points, with an AUC of 0.85, 76.5% sensitivity, and 89.3% specificity.

**Conclusion:**

The SSS-8 is a useful indicator for SSRD clinical outcomes. Patients with SSRD may need an SSS-8 score decrease of 6 or more points to notice symptom improvements.

## Main Points

1. Longitudinal changes in Somatic Symptom Scale-8 (SSS-8) scores were shown to adequately reflect subjective improvement in patients with somatic symptoms and related disorders (SSRD) at follow-up.

2. Receiver operating characteristic analyses revealed that a decrease of 6 or more points in the SSS-8 score suggested a clinically meaningful change in patients with SSRD.

3. As improvement in subjective symptoms is an important treatment goal for SSRD, the SSS-8 was found to be a useful indicator for assessing the clinical outcomes of SSRD.

## 1. Introduction 

Patients with somatic symptoms and related disorders (SSRD) [1] are extremely focused on their chronic physical symptoms. However, tests rarely reveal abnormalities consistent with their symptoms [2]. Consequently, these patients often visit doctors to request tests that are not medically necessary [3]. Since SSRD is not associated with fatal organic abnormalities, clinicians tend to downplay this condition [4]. SSRD symptoms become fixed over time [5], and if patient-perceived symptoms persist for more than 3 months, they may still be present 5 years later [6]. The most unfulfilled expectation for patients with SSRD is the prognosis [7] and clinicians need to clarify the rationale for the outcome and prognosis after treatment.

Although it is not easy to quantify the diverse clinical manifestations of SSRD, questionnaires such as the Somatic Symptom Scale-8 (SSS-8) [8] are used because SSRD status is primarily assessed on the basis of the subjective symptoms. The SSS-8, comprising 8 questions, is a simplified version of the Patient Health Questionnaire-15 [9], which enables a more rapid assessment of the status of SSRD. A method using the SSS-8 as a screening tool to determine SSRD severity according to cutoff values has been reported [10]. SSS-8 scores could serve as quantitative markers of somatic symptoms, and iterative application of the SSS-8 can be used to monitor the somatic symptom burden and reevaluate the patient’s medical status [8].

The minimal clinically important difference (MCID) [11] is a concept that describes the smallest amount of change in the patient’s condition that is perceived as a significant improvement sufficient enough to be clinically relevant. Using the MCID, anchor questions that accurately reflect changes in the patient’s condition are usually used to estimate improvement thresholds. Questionnaires have been used clinically not only to screen physical and mental problems but also as indicators of changes in the medical condition. However, to date, no study has established the MCID of the SSS-8. Therefore, this study aimed to clarify the MCID value of the SSS-8 for SSRD.

## 2. Materials and Methods

### 2.1 Participants

This longitudinal study recruited patients with SSRD aged 18 to 84 years who visited the Department of Psychosomatic Medicine, Toho University Medical Center Omori Hospital between November 10, 2023, and August 21, 2024.

The exclusion criteria were: (1) schizophrenia spectrum disorders and other psychotic disorders; (2) dementia (such as Alzheimer’s, vascular, Parkinson’s disease, and Lewy body dementia); (3) suicidal ideation; (4) Japanese was not the first language; and (5) patients who could not be accurately assessed because of missing or incorrect responses.

All patients responded to self-report questionnaires at baseline and at the 6-month follow-up. Participants took approximately 15 min to respond to the questionnaire forms. Participants’ demographic characteristics such as age, sex, alcohol consumption history, smoking history, educational duration, disease duration, treatment history, and comorbid symptoms were assessed. Participants’ physical symptoms were reassessed approximately 6 months after the initial assessment, and the magnitude of change was evaluated. In addition, participants underwent evaluations of the subjective outcomes of symptoms. All participants continued to receive outpatient treatment during this period.

The primary endpoint was assessed using the Patient Global Impression of Change (PGIC) [12], whose component questions were set as the anchor questions. The secondary endpoint was assessed using the subscale pertaining to physical function from the Multidimensional Evaluation Scale for Patient Impression Change (MPIC) [13], whose component questions were set as the anchor questions, similar to the primary endpoint. Two experts unanimously diagnosed all the participants with SSRD, whose diagnostic criteria were based on the Diagnostic and Statistical Manual of Mental Disorders, Fifth Edition, Text Revision [1].

### 2.2 Questionnaires

Changes in participant physical symptoms were assessed using the SSS-8. The SSS-8 is a scale of 8 general symptoms including “chest pain or shortness of breath” and “headaches”, which are rated along a 5-point scale ranging from “0 (not at all)” to “4 (very much)”; higher scores indicate more severe somatic symptoms. The Japanese version of the SSS-8 [14] has been validated for linguistic, psychological, and internal consistency [15]. Cronbach’s alpha for the Japanese version of the SSS-8 was found to be 0.86 [15].

The PGIC is scored using a 7-point Likert-type scale from 1 = “Very Much Improved” to 7 = “Very Much Worse”. As the purpose of this study was to identify clinically definite changes in physical symptoms, a score of 1 or 2 on the PGIC was designated as a clear improvement in physical symptoms.

The MPIC is an expansion of the PGIC [12], and includes 7 additional domains (Pain, Mood, Sleep, Physical Functioning, Cope with Pain, Manage Pain Flare-ups, and Medication Effectiveness), and each of the 8 domains can be used as an independent scale [13]. Cronbach’s alpha value was 0.89 [13, 16]. The MPIC is also scored along a 7-point Likert-type scale from 1 = “Very Much Improved” to 7 = “Very Much Worse”, and the Japanese version is evaluated in the same way [16]. In this study, a clear improvement in physical function was defined as a score of 1 or 2 on the MPIC Physical Functioning scale.

Participants also underwent initial assessment for anxiety and depressive symptoms [17] as these psychological conditions can co-exist with SSRD. The Hospital Anxiety and Depression Scale (HADS) [18] consists of anxiety and depression scales. Each scale is scored from 0 to 21, with higher scores indicating more intense symptoms. The Japanese version of the HADS was validated for reliability and validity [19] and factor structure [20] by a Japanese study. Cronbach’s alpha coefficients for the HADS-A and HADS-D were 0.81 and 0.76, respectively [21].

### 2.3 Data Analyses

The demographic characteristics of the participants in the improved and non-improved groups were compared. In the analysis of the primary endpoint, given the sample size and expectations, sex, marital status, smoking and drinking habits, and medical therapy (antidepressant and/or benzodiazepine use) were compared using the chi-squared test. Medical therapy: antipsychotic use was compared using the Fisher exact test. Given the normality and variance of the data, Student’s *t*-test was used to compare the age and scores on each questionnaire, and the Mann–Whitney U test was used to compare educational history and disease duration.

In the analysis of the secondary endpoint, sex, marital status, drinking habit, and medical therapy (antidepressant and/or benzodiazepine use) were compared using the chi-squared test. Medical therapy (antipsychotic use) and smoking habit were compared using the Fisher exact test. Given the normality and variance of the data, Student’s *t*-test was used to compare the age and scores on each questionnaire, and the Mann–Whitney U test was used to compare educational history and disease duration. Normally distributed data were presented as the mean and standard deviation (SD), and non-normally distributed data as the median and interquartile range.

Thereafter, receiver operating characteristic (ROC) analyses [22] were used to calculate the MCID values of the SSS-8 for each of the primary and secondary endpoints to identify those with clear improvements in physical symptoms. The MCID cutoff point was identified by calculating the Youden index [(sensitivity + specificity) – 1] to maximize sensitivity and specificity [23].

EZR (“Easy R”) software program, version 1.54 (Saitama Medical Center, Jichi Medical University, Saitama, Japan) [24] was used for all analyses, and two-tailed *p*-values < 0.05 were considered statistically significant.

## 3. Results

**Fig. 1.**
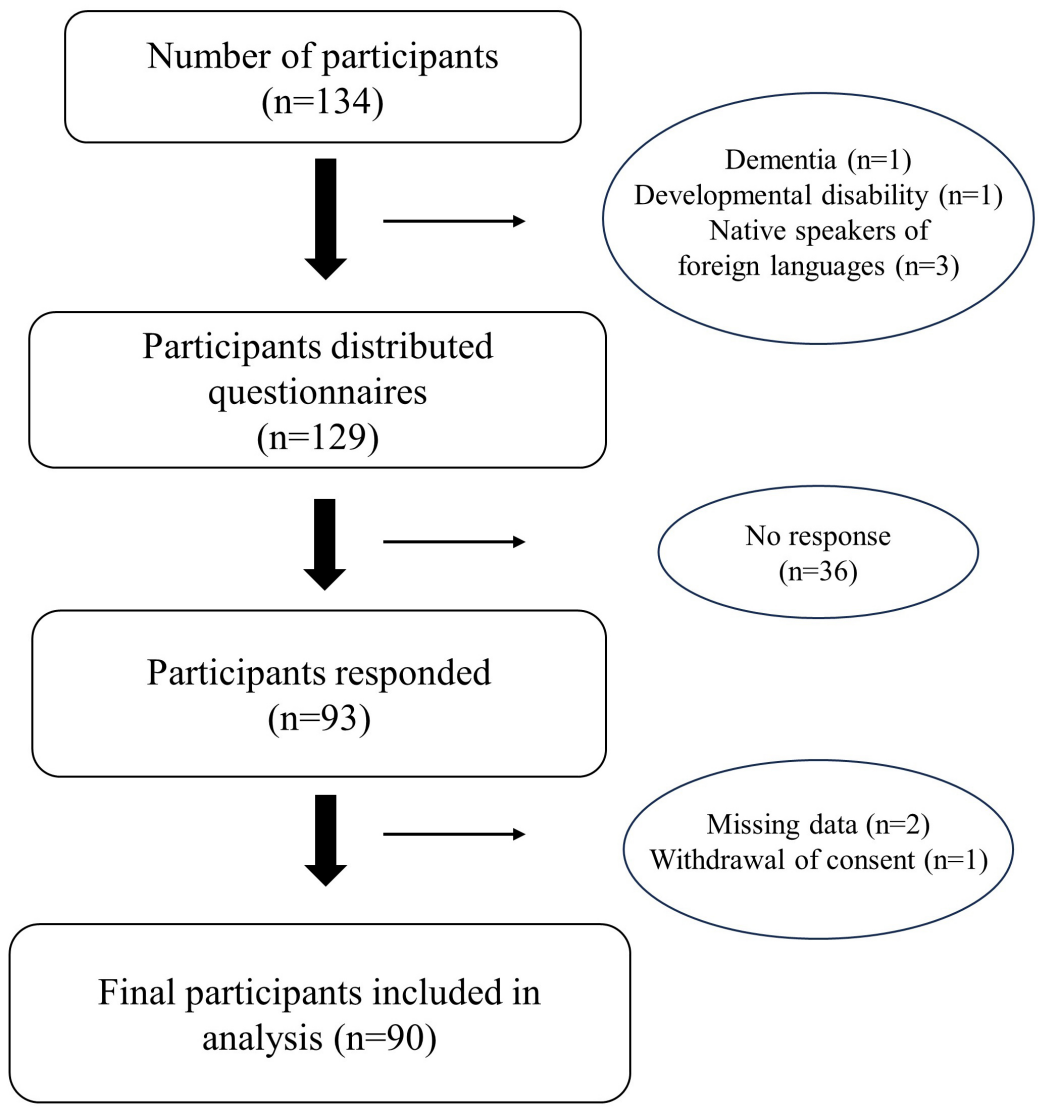
**Flowchart of patient enrollment**.

Of the 134 participants, 1 patient with dementia, 1 patient with a developmental disability, and 3 patients whose first language was not Japanese were excluded. Thus, a total of 129 questionnaires were distributed, and 93 participants responded (response rate 72.1%). Of these, data were missing for 2 patients and 1 withdrew consent to participate. Finally, 90 participants were included in the analysis (Fig. 1). The mean age of the 90 participants was 49.7 ± 16.3 years, of whom 31 were men and 59 were women. The median morbidity duration of the participants was 21 [15.3–37.5] months, and the mean initial SSS-8 score was 16.4 ± 5.5 points.

Tables 1,2 present the comparisons of the demographic characteristics for the improved and non-improved groups. When grouped by the primary endpoint, 44 participants were assigned to the improvement group and 46 to the non-improvement group. No clear differences were detected between the two groups with respect to most demographic characteristics, such as age, sex, marital status, disease duration, pharmacotherapy introduced, or in the scores on the various questionnaires; however, the proportion of smokers was 4.5% in the improved group, which was significantly lower than that in the non-improved group (*p* < 0.05). Thereafter, when stratified by the secondary endpoint, 34 participants were assigned to the improvement group and 56 to the non-improvement group. The improved group had a median of 15.0 years of education, which was significantly longer than the non-improved group (*p* < 0.05). The proportions of smokers and benzodiazepine users were 2.9% and 35.3%, respectively, in the improved group, both of which were significantly lower than those in the non-improved group (*p* < 0.05). Other demographic characteristics did not differ between the two groups.

**Table 1.** **Comparison of characteristics of participants in the primary endpoint (n = 90)**.

		Improved (n = 44)	Non-improved (n = 46)	*p* value
Sex				0.945^a^
	Male	15 (34.1%)	16 (34.8%)	
	Female	29 (65.9%)	30 (65.2%)	
Age: years (mean ± SD)		50.8 ± 17.0	48.6 ± 15.7	0.515^c^
Habit				
	Smoke	2 (4.5%)	9 (19.6%)	0.030^a^
	Drink	12 (27.3%)	11 (23.9%)	0.715^a^
Marriage		28 (63.6%)	24 (52.2%)	0.271^a^
Education: year (median [Q1–Q3])		14.0 [12.0–16.0]	12.0 [12.0–16.0]	0.152^d^
Morbidity duration: month (median [Q1–Q3])		20.0 [13.8–39.0]	24.0 [18.0–37.5]	0.394^d^
Medical therapy				
	Antidepressant	30 (68.2%)	26 (56.5%)	0.254^a^
	Antipsychotics	3 (6.8%)	6 (13.0%)	0.486^b^
	Benzodiazepines	18 (40.9%)	27 (58.7%)	0.092^a^
Questionnaire				
	Somatic Symptom Scale-8: initial measurement (mean ± SD)	17.4 ± 5.5	15.3 ± 5.3	0.063^c^
	Somatic Symptom Scale-8: second measurement (mean ± SD)	9.0 ± 4.0	14.8 ± 4.7	<0.001^c^
	Hospital Anxiety and Depression Scale Anxiety (mean ± SD)	11.7 ± 4.7	9.8 ± 4.9	0.064^c^
	Hospital Anxiety and Depression Scale Depression (mean ± SD)	10.8 ± 5.0	9.7 ± 5.3	0.285^c^

^a^: Chi-squared test. ^b^: Fisher exact test. ^c^: Student’s *t*-test. ^d^: Mann–Whitney U test. SD, standard deviation; Q1, first quartile; Q3, third quartile.

**Table 2.** **Comparison of characteristics of participants in the secondary endpoint (n = 90)**.

		Improved (n = 34)	Non-improved (n = 56)	*p* value
Sex				0.090^a^
	Male	8 (23.5%)	23 (41.1%)	
	Female	26 (76.5%)	33 (58.9%)	
Age: years (mean ± SD)		50.3 ± 16.2	49.6 ± 16.5	0.775^c^
Habit				
	Smoke	1 (2.9%)	10 (17.9%)	0.047^b^
	Drink	8 (23.5%)	15 (26.8%)	0.731^a^
Marriage		22 (64.7%)	30 (53.6%)	0.300^a^
Education: year (median [Q1–Q3])		15.0 [12.0–16.0]	12.0 [12.0–16.0]	0.018^d^
Morbidity duration: month (median [Q1–Q3])		20.5 [15.3–36.0]	21.5 [15.5–40.5]	0.963^d^
Medical therapy				
	Antidepressant	25 (73.5%)	31 (55.4%)	0.085^a^
	Antipsychotics	3 (8.8%)	6 (10.7%)	0.999^b^
	Benzodiazepines	12 (35.3%)	33 (58.9%)	0.030^a^
Questionnaire				
	Somatic Symptom Scale-8: initial measurement (mean ± SD)	17.6 ± 5.5	15.6 ± 5.3	0.082^c^
	Somatic Symptom Scale-8: second measurement (mean ± SD)	8.3 ± 4.1	14.2 ± 4.6	<0.001^c^
	Hospital Anxiety and Depression Scale Anxiety (mean ± SD)	11.4 ± 4.5	10.4 ± 5.1	0.322^c^
	Hospital Anxiety and Depression Scale Depression (mean ± SD)	11.0 ± 4.7	9.8 ± 5.4	0.292^c^

^a^: Chi-squared test. ^b^: Fisher exact test. ^c^: Student’s *t*-test. ^d^: Mann–Whitney U test.

**Fig. 2.**
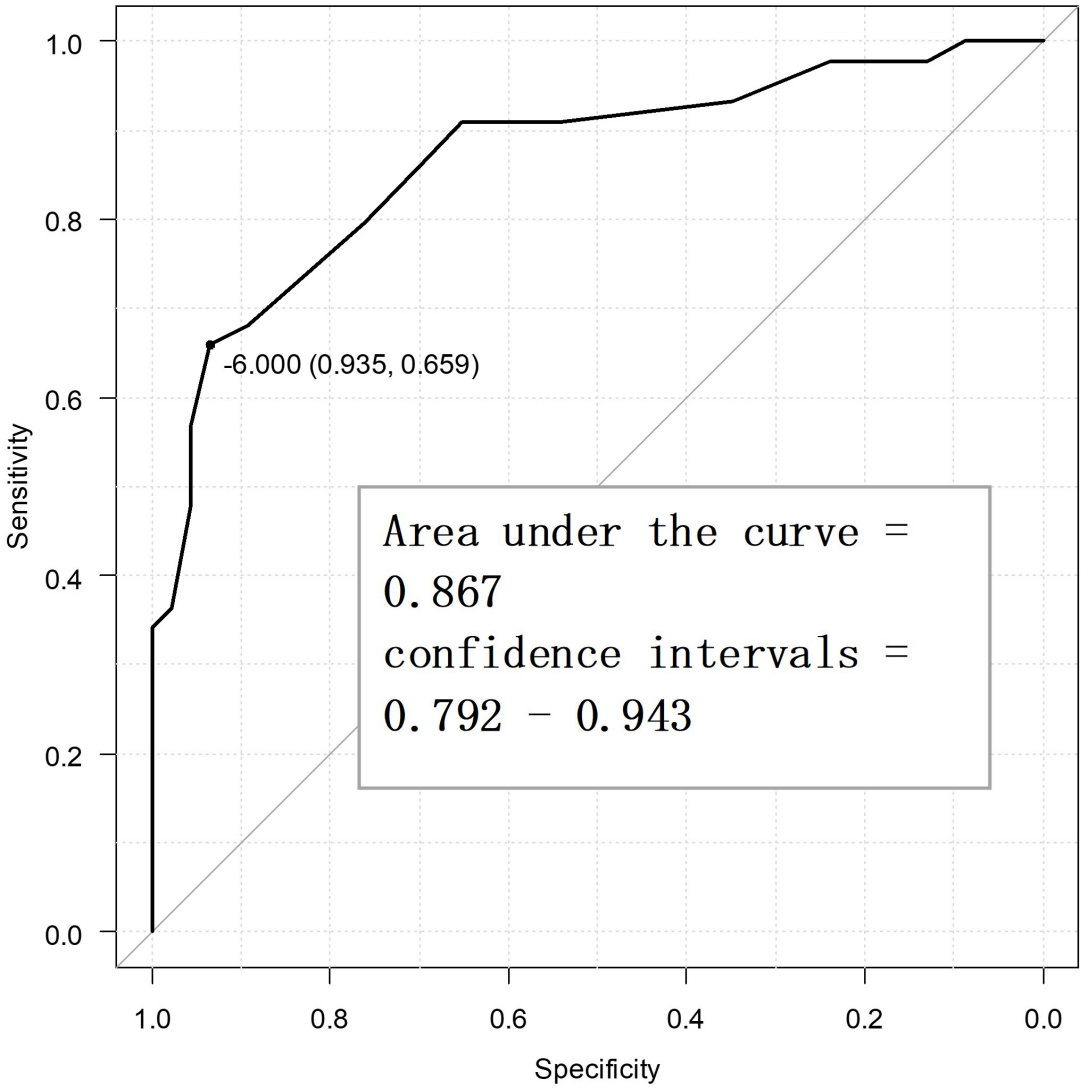
**Receiver operating characteristic curve for the primary endpoint to determine the minimal clinically important difference points (n = 90)**.

**Fig. 3.**
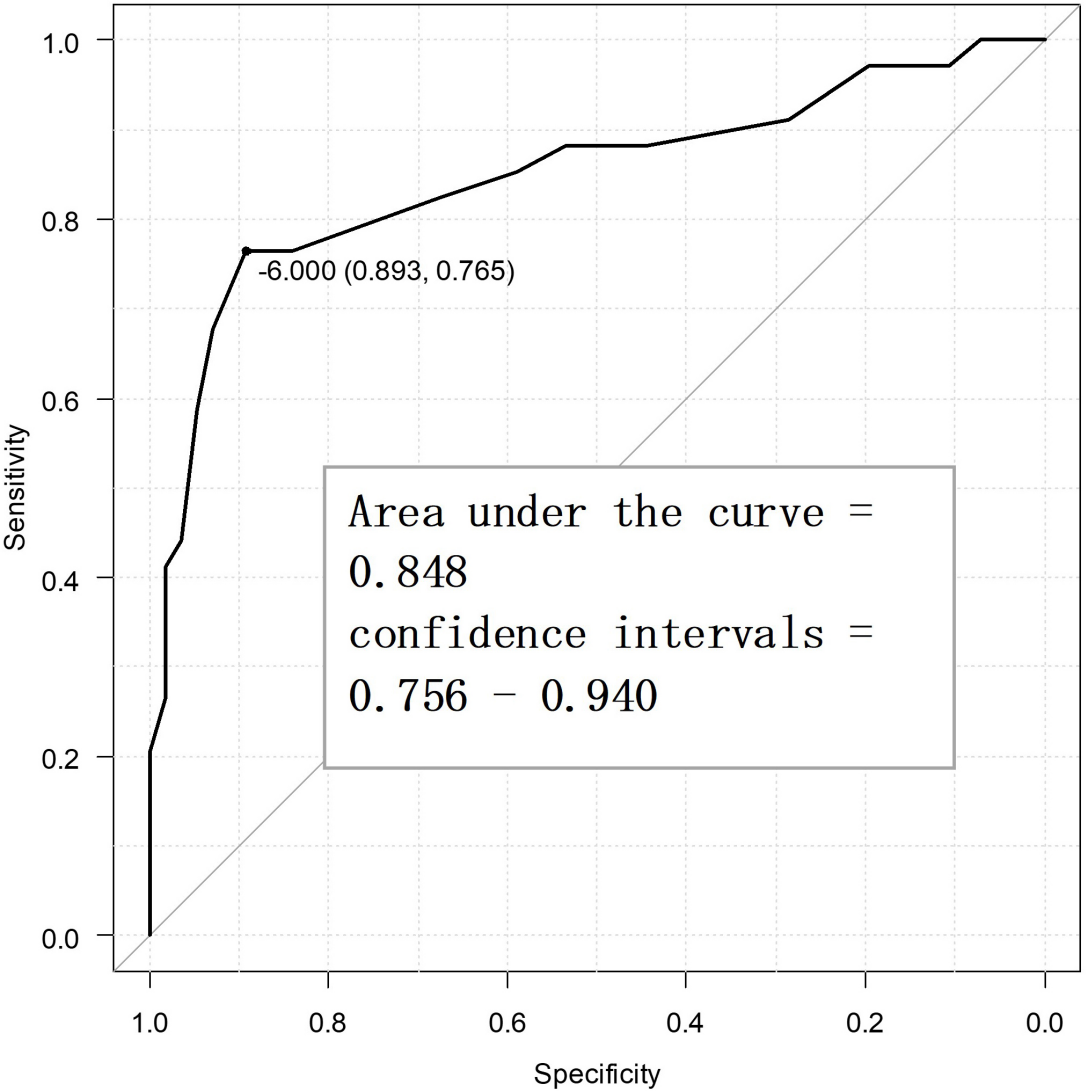
**Receiver operating characteristic curve for the secondary endpoint to determine the minimal clinically important difference points (n = 90)**.

Fig. 2 displays the ROC curve for the primary endpoint, viz. PGIC. The optimal cutoff value for the SSS-8 using the Youden index was –6 points, while the sensitivity was 65.9%, specificity was 93.5%, and the AUC was 0.867 (confidence interval = 0.792–0.943). Fig. 3 displays the ROC curve for the secondary endpoint, i.e., the MPIC physical function subscale. The optimal cutoff value of the SSS-8 using the Youden index was –6 points, while the sensitivity was 76.5%, specificity was 89.3%, and AUC was 0.848 (confidence interval = 0.756–0.940). In other words, the MCID value of the SSS-8 was –6 points for both the primary and secondary endpoints.

## 4. Discussion

### 4.1 Interpretation of Results

In this study, we calculated the MCID value of the SSS-8 to develop criteria for assessing the clinical outcomes of patients with SSRD. The optimal SSS-8 MCID for the PGIC and MPIC physical function subscale was –6 points. Our results suggest that the SSS-8 possesses potential for assessing the clinical outcomes of somatic symptoms. A reduction of 6 or more points in the SSS-8 score was interpreted as a clinically meaningful change.

### 4.2 Comparison With Existing Literature

Most patients with somatic and related syndromes have a high degree of functional disability [25], although no abnormalities are found upon biological examination [3]. The SSS-8 is used as an adjunctive screening tool for diagnosis [1] and determining the clinical severity [8, 10]. According to a German study, the SSS-8 represents five levels of severity in 4-point increments [8]. Assessing disease severity is important for patients and clinicians, as each stage of the illness indicates different clinical symptoms and numbers of health care visits. A previous study showed that a shorter educational duration was associated with lower treatment responsiveness [26], which is consistent with the results of the present study. Additionally, studies have shown that smokers are more prone to pain [27], somatization, and psychiatric problems [28], which may explain the large number of smokers in the non-improvement group for physical function.

### 4.3 Study Limitations

This study had some limitations. First, numerous patients had anxiety and depressive symptoms, making it difficult to separate the effects of the comorbidities in SSRD. Second, because of the lack of established treatment for SSRD, we could not eliminate the possibility that the lack of uniformity in the treatment approach could potentially have affected the results. Third, since we did not consider differences in disease severity, we could not determine whether the significance of a change in 6 or more points differed between participants with high and low SSS-8 scores. Fourth, the AUC values in this study indicated moderate diagnostic accuracy [29], but our interpretation of the longitudinal results was limited because the SSS-8 was developed as a screening instrument basically. Finally, although the participants exhibited characteristics of common SSRD such as a high female preponderance and longer chronic disease duration [1], the sample size was limited because of its single-center setting, and the participants’ educational level may have been higher than that of patients with general SSRD, limiting the generalizability of the results.

### 4.4 Practical Implications

It would be meaningful for clinicians to visualize the clinical course of SSRD using the SSS-8 for quantitative evaluation. Since improvement in subjective symptoms is an important treatment goal for SSRD, a six-point or greater reduction in the SSS-8 scores shown in this study can be used as a clinical indicator. However, the results suggest that patients with SSRD may not perceive a clear improvement in their subjective symptoms when severity is improved by merely one level [8], reflecting excessive health consciousness among this population.

Since pharmacotherapy for SSRD has not been established, benzodiazepines may be used to mitigate physical symptoms by providing emotional relief, although there is a risk of dependence [30]. Even though the causal relationship is unknown, the persistence of physical symptoms in the non-improvement group may have promoted the use of benzodiazepines as symptomatic treatment.

### 4.5 Future Research Directions

This study could serve as a clinical benchmark for patients with SSRD and inform future research to establish treatment plans. Future research should consider differences in disease severity and control treatments that participants receive to rule out the possibility that differences in treatment methods may affect subjective symptoms. Additionally, larger, multi-center studies are warranted to expand the generalizability of the results and stratify participants by background factors.

## 5. Conclusion

To estimate clinically significant changes in disease status for SSRD, the MCID values for the SSS-8 scores were determined. The SSS-8 is a useful indicator of clinical outcomes for SSRD. Patients with SSRD may perceive a clear improvement in their symptoms if their SSS-8 score decreases by 6 or more points.

## Availability of Data and Materials

We are not able to share our data because sharing data is not permitted by our hospital ethics committees. But, the data supporting the findings of this study are available on reasonable request from the corresponding author.
